# Role of the G Protein-Coupled Receptor, mGlu_1_, in Melanoma Development

**DOI:** 10.3390/ph3092821

**Published:** 2010-08-26

**Authors:** Janet Wangari-Talbot, James Goydos, Suzie Chen

**Affiliations:** 1 Graduate School of Biomedical Sciences-Robert Wood Johnson Medical School, Piscataway, 08854 New Jersey, USA; 2 Department of Surgery, Cancer Institute of New Jersey, New Brunswick, 08901 New Jersey, USA; 3 Susan Lehman Cullman Laboratory for Cancer Research, Ernest Mario School of Pharmacy, Rutgers, The State University of New Jersey, Piscataway, 08854-8020 New Jersey, USA

**Keywords:** GPCR, Melanoma, *GRM1*, mGlu_1_

## Abstract

Melanoma remains one of the cancers for which a decline in morbidity has not been achieved with current scientific and medical advances. Mono-therapies targeting melanoma have been largely ineffective, increasing the need for identification of new drugable targets. Multiple tumor suppressors and oncogenes that impart genetic predisposition to melanoma have been identified and are being studied in an attempt to provide insight on the development of anti-melanoma therapies. Metabotropic Glutamate Receptor I (*GRM1*) has recently been implicated as a novel oncogene involved in melanomagenesis. *GRM1* (mGlu_1_, protein) belongs to the G protein coupled receptor (GPCR) super family and is normally functional in the central nervous system. Our group showed in a transgenic mouse model system that ectopic expression of *Grm1* in melanocytes is sufficient to induce spontaneous melanoma development *in vivo*. GPCRs are some of the most important therapeutic drug targets discovered to date and they make up a significant proportion of existing therapies. This super family of transmembrane receptors has wide spread expression and interacts with a diverse array of ligands. Diverse physiological responses can be induced by stimulator(s) or suppressor(s) of GPCRs, which contributes to their attractiveness in existing and emerging therapies. GPCR targeting therapies are employed against a variety of human disorders including those of the central nervous system, cardiovascular, metabolic, urogenital and respiratory systems. In the current review, we will discuss how the identification of the oncogenic properties of *GRM1* opens up new strategies for the design of potential novel therapies for the treatment of melanoma.

## Introduction

Skin cancer is one of the most common types of cancer in the United States, with an estimated 1 in 5 Americans developing some form of skin cancer in their lifetime [1]. Melanoma, the most deadly form of skin cancer, is a malignant tumor derived from epidermal melanocytes and can occur in any melanocyte-containing tissue such as the eyes, oral mucosa, nasopharynx, trachea-bronchial tree, vulva, vagina, anus, urinary tract, central nervous system (CNS) and most commonly, the skin [2]. The American Cancer Society (ACS) estimates that in 2010 ~68,130 new cases of cutaneous melanoma will be diagnosed in the United States, with ~8,700 fatalities occurring [3]. The ACS also estimates the lifetime overall risk for developing melanoma to be 1 in 50 in Caucasians, 1 in 1,000 in blacks and 1 in 200 in Hispanics. Early detection, surgery and adjuvant therapy have led to improved outcomes in the management of melanoma, but poor prognosis for those with metastatic disease still persists. The survival rate for patients with advanced metastasis ranges from 2 to 8 months, with survival rates of ~5% after 5 years [4]. The clinical and histopathological aspects of melanoma development and progression are already well established, whereas the molecular mechanisms of the disease continue to unravel with the availability of high-throughput molecular technologies within the last few years. This has allowed scientists to pinpoint melanoma predisposition candidate genes that include oncogenes and tumor suppressors. Loss of cell cycle regulation through mutations in the *p16INK4A* gene accounts for ~20% of familial melanoma [5,6,7]. *p16INK4A* inhibits the G1-S transition by blocking the activity of cyclin-dependent kinases 4 and 6 (*CDK4* and *CDK6*) that phosphorylate and inactivate the Rb tumor suppressor [6,8,9]. Mutations in *p16INK4A* interfere with binding to *CDK4/CDK6* and thus the ability to limit Rb phosphorylation and cell-cycle progression [6,8,9,10]. Other predominant mutations identified in melanoma development are components of the MAPK signaling cascade, RAS-RAF-MEK-ERK. Activating mutations of the *BRAF* gene have been found in ~70% of melanomas, in particular, the BRAF V600E mutation, which is also found in nevi that are thought to be pre-malignant lesions [11,12,13,14,15,16]. RAF kinases are known to activate the MAPK signaling pathway after activation by GTP bound RAS [12,13,14,15]. Activated RAF activates the MEK kinase, which then phosphorylates and activates ERK, which has multiple downstream targets and results in alterations in gene transcription to promote cell proliferation and resistance to apoptosis. The N-RAS mutation is the most common of *RAS* gene mutations particularly at residue 61 and maintains the protein in a constitutively activated state [17]. Mutated N-RAS has been detected in 15–30% of melanomas [17,18]. In addition to the contributions by these well-known genes in melanomagenesis, other putative genes continue to be discovered. Our group has identified the oncogenic potential of a neuronal GPCR, mGlu_1,_ when it is ectopically expressed in melanocytes [19].

## GPCRs

GPCRs are among the largest and most diverse family of proteins in the mammalian genome which transduce signals as a response to a wide range of stimuli. GPCRs are major targets in drug discovery, as reflected by the fact that they encompass about 50% of current medicinal compounds [20]. GPCRs are evolutionarily conserved and have been identified in multiple species [21,22]. In humans, the completed human genome project has led to the identification of over 865 GPCR genes [23]. The diversity of GPCRs is dictated not only by the variety of stimuli that they respond to, but also their participation in various signaling pathways. Ligands for these receptors are diverse including light, odorants, neurotransmitters, hormones, peptides and nucleotides [20]. 

## Role of GPCRs in Human Diseases

The importance of GPCRs in drug discovery results from their widespread expression, especially on the cell surface, that makes them accessible to antagonists, agonists, hormones and drugs, as well as tissue and cell type specificity, which provides selectivity for the receptors and ligands [24]. Exploration of various drug targets has lead to the identification of multiple ways in which GPCRs contribute to a disease state. Classification of GPCR related diseases fall into categories of either rare monogenic disease resulting from loss or gain of function mutations in GPCRs, from genetic variants of GPCRs or from defects in G proteins [25,26,27]. A well studied monogenic disease caused by a GPCR is nephrogenic diabetes insipidus (NDI). NDI results from a failure of the anti-diuretic hormone, vasopressin to act on the renal collecting duct to facilitate water re-absorption due to mutations in the arginine vasopressin receptor 2 (AVPR2) [25,26,28]. These loss-of-function mutations prevent the transmembrane receptors from activating G proteins and the effector adenylyl cyclase by interfering with the folding and insertion of the receptor into the plasma membrane [28]. Genetic variants or polymorphism of various GPCRs have also been implicated in human disease. In congestive heart failure, the combination of the polymorphic alpha2C-adrenergic receptor and a variant of the beta1-adrenergic receptor synergize to increase the release of norepinephrine and increase receptor activity leading to the development and progression of heart failure [29]. In vitiligo, a disease characterized by the loss of melanocytes resulting in cutaneous white macules, a study in a subset of Korean patients found them to have more polymorphisms than those with no vitiligo in the GPCR melanocortin 1 receptor (MC1R) which controls melanomagenesis even though the finding was not statistically significant [30]. The polymorphisms in GPCRs can also have a protective effect against infections as observed in HIV. In studies examining HIV infection resistance in people with multiple exposures to the virus, homozygous loss-of-function mutations of the type 5 chemokine receptor (CCR5) were found to confer resistance to HIV infection as CCR5 serves as a co-receptor for HIV entry into the target cell [31]. Defects in G proteins especially Gα subunits (transducin and G_s_α) are also associated with human diseases. Mutations in transducin causes it to uncouple from its effector and has been associated with the Nougaret form of autosomal dominant stationary night blindness [27]. Mutations in Gβ or Gγ have not been associated with any monogenic human disorders to-date, but a polymorphism of the β_3_ subunit has been implicated in several common multigenic disorders [26].

## GPCRs as Oncogenes

Genetic alterations that change the signaling activities and expression patterns of oncogenes and tumor suppressors have been shown to result in various human cancers. GPCRs are involved in the transduction of cellular signals that govern cell proliferation, apoptosis and metastasis. Maintaining a balance between normal cell growth and aberrant cell growth is critical to prevent a normal cell from exhibiting a cancerous phenotype [32]. Potent mitogens such as vasopressin, angiotensin II and acetylcholine receptor agonists have been shown to stimulate their cognate receptors leading to cellular proliferation [33,34,35]. Moreover, autocrine and paracrine signaling induced by GPCR ligands and agonists has also been shown to stimulate tumor growth in numerous cancers. In small cell lung cancer, gastrin-releasing peptide [36] and neuromedin B [37] are secreted and released by the tumor cells, the majority of which express receptors to these ligands. Autocrine signaling has also been observed in melanoma cells that express mGlu_1_ and are dependent on glutamate to promote their proliferation and thus secrete more glutamate [38] than normal human melanocytes [38,39]. A link between GPCRs, cellular proliferation and oncogenesis was established with the discovery of a novel oncogene, *mas* [40], which was capable of transforming mouse fibroblasts. DNA from a human epidermal carcinoma was transfected into NIH 3T3 fibroblasts resulting in cells with weak foci-forming abilities *in vitro* but was highly tumorigenic in nude mice in the absence of activating mutations*.* This novel proto-oncogene was found to have seven distinct hydrophobic domains which were predicted to be transmembrane domains suggesting that *mas* was a membrane spanning integral protein. Further studies showed that other wild type GPCRs could be tumorigenic when ectopically expressed and exposed to their cognate ligands [34,41]. Julius *et al.* [41] demonstrated that the neuronal restricted expression of the serotonin receptor 5HT1c when ectopically expressed in fibroblasts altered the growth phenotypes of the fibroblasts by induction of dense foci formation in cultured cells and tumorigenicity in nude mice. Subsequent to this study, Gutkind *et al*. [34] also showed that the ectopic expression and activation of the M_1_, M_2_, M_3_, M_4_ and M_5 _GPCR subtypes of the muscarinic acetylcholine receptor family (mAChR) by their agonists in fibroblasts also led to cellular transformation. This study also yielded similar results to those of Cuttitta [36] and Cardona [37] that showed the induction of transforming potential of normal GPCRs when exposed to high concentrations of a cognate ligand. The Kaposi sarcoma-associated herpes virus G protein coupled receptor (KSHV-GPCR) was also shown to promote angiogenesis by inducing the expression and secretion of VEGF when it is ectopically expressed in mouse fibroblasts [42]. This angiogenic activity is independent from its oncogenic action in Kaposi sarcoma, where it promotes cellular proliferation through its stimulation of protein kinase B/Akt [43,44]. More recently, the ectopic expression of the gastric inhibitory peptide receptor (GIPR) in adrenal cortical cells has been shown to be sufficient to induce adrenal hyperplasia and adenomas associated with Cushing’s syndrome [45,46]. It is now clear that there is an undeniable link between the ectopic expression of wild type GPCRs and various human cancers. Our group has contributed to the understanding of the oncogenic potential of GPCRs. We have established the etiological role of *GRM1* in melanoma. *GRM1* is a gene normally expressed and functional in the CNS, however, when *GRM1* is ectopically expressed in mouse or human melanocytes, one of the consequences is cellular transformation *in vitro* and tumor formation *in vivo* [19].

## Metabotropic Glutamate Receptors

Glutamate is the major excitatory neurotransmitter in the central nervous system and its signaling has been shown to be mediated by the glutamate receptor class of GPCRs [47]. Initially, it was thought that synaptically released glutamate was only involved in the opening of cation permeable channels through the action of ionotropic glutamate receptors; however, subsequent studies have shown that glutamate can also induce the hydrolysis of phosphoinosites [48] or decrease adenylyl cyclase activity through G protein coupled metabotropic glutamate receptors [49]. Metabotropic glutamate receptor 1 was initially identified and sequenced in 1991 [49,50]. Since then, seven other metabotropic glutamate receptors have been recognized. These eight metabotropic glutamate receptors have been characterized and categorized into three groups, based on agonist pharmacology, sequence homology and transduction mechanisms via coupling to second messenger systems [48,49,50,51]. Group I metabotropic glutamate receptors includes mGlu_1_ and mGlu_5_ which are predominantly located in post synaptic elements. They couple primarily to G_q_ proteins to increase phosphoinositide hydrolysis via activation of the phosholipase C pathway, which also results in intracellular calcium release [52,53]. Group II metabotropic glutamate receptors include mGlu_2_ and mGlu_3_ and they are found in both presynaptic and post synaptic elements. mGlu_2_ and mGlu_3_ couple to G_i_/_0_ proteins and mediate downstream signaling through the adenylyl cyclase inhibition systems. Group III metabotropic glutamate receptors are mGlu_4_, mGlu_6_, mGlu_7_ and mGlu_8_ and they are expressed and functional in presynaptic elements and similar to Group II mGlus they also couple to G_i_/_0_ proteins [51,52,53]. Upon stimulation of a GPCR by its ligand, a G protein couples the activated receptor to its effector leading to intracellular signaling. G proteins are heterotrimeric proteins that consist of α and β/γ subunits. These subunits are associated with one another only when bound to GDP (inactive state). Activation of the GPCR by its ligand leads to the exchange of GDP for GTP and the dissociation of Gα-GTP subunit from the Gβ/γ, both of which can act as independent signaling molecules [32,54]. 

## mGlu_1_ in Melanoma

Mouse models provide an invaluable tool in tumor biology. In melanoma, numerous transgenic mouse models have been generated using transgenes expressed under the control of the melanocyte-specific tyrosinase promoter, including the SV40 early region transforming sequences [55] and activated *RAS* [56,57]. Ubiquitously expressed transgenes such as *RET* [58] and hepatocyte growth factor [59] have also been used to induce melanomagenesis when expressed under a mouse metallothionein promoter. Other transgenic mouse models require the utilization of a carcinogenic insults such as 7,12-dimethylbenz(a)anthracene (DMBA) and 12-*O*-tetradecanoylphorbol-13-acetate (TPA) to induce and promote melanoma formation [9,60,61]. A major drawback of these models is that the development of melanoma has a long latency period and frequently requires more than one carcinogen to induce tumors which tend to metastasize at very low rates or are not metastatic at all [9,60,61]. This, in addition to the development of other neoplasms such as fibrosarcomas, papillomas, and squamous cell carcinomas complicates the utility of these models for the study of the onset and progression of melanoma. Our group has generated a mouse model that spontaneously develops melanocytic lesions that progress into invasive or metastatic tumors with short latency in the absence of known carcinogenic insults or other tumor types [62].

We generated five transgenic founder mice using Clone B [63], a small 2 Kb genomic DNA fragment previously shown to commit mouse fibroblasts into adipocyte differentiation [62,64]. Each of these transgenic lines was found to have 5-7 copies of the transgene integrated into different regions of their genome [62]. The expected obese phenotype was not observed in any of the transgenic lines, rather, one of the transgenic founder mice (TG3), developed pigmented lesions on several externally visible sites including the eyes, ears, snout, skin and peri-anal region at 8 months of age, while the other four founder mice appeared normal [62]. These melanocytic lesions increased in size and number as the founder mouse aged. Histological evaluation of the pigmented lesions revealed them to be melanoma. Subsequent progeny of this founder mouse also exhibited external pigmented lesions at a young age (3–4 months old). This founder mouse was sacrificed at 14 months due to tumor burden. Necropsies performed on this founder mouse established pigmented lesions in the lymph nodes, brain, muscles, lungs, choroid plexus, harderian gland of the eye and inner ears. Histologically these lesions resembled those observed in human melanomas [62]. Further analyses in young post natal mice from post natal day (PND) 1 to PND 30 showed that as early as PND 1, the transgenic mice had twice the number of melanocytes as the non-transgenic mice, moreover by PND 15; the number of melanocytes in transgenic mice was 11 times that of the wild type littermates. Both transgenic and non-transgenic mice displayed a decrease in the number of inter-follicular melanocytes at PND 7 due to the migration of the melanocytes to the hair follicles [65]. Also at PND 7, melanocytes were morphologically indistinguishable between TG3 and wild type littermates, however, unlike the wild type littermates, the number of epidermal melanocytes increased in TG3 mice instead of remaining constant or even decreasing in the skin. In TG3 mice at PND 15, clusters of melanocytes likely derived from clonal expansion were noted. By PND 30, large rounded and heavily pigmented melanocytes that resembled those observed in adult TG3 mice were noted on the skin, choroid plexus and harderian gland of the eye supporting the notion that the origin of these pigmented lesions were neural crest derived melanocytes [65]. From genome mapping studies on the TG3 mouse line, we determined that there was only one transgene-integration event on mouse chromosome 10. This integration event resulted in a deletion of about 70kb of the host sequence. This region of mouse chromosome 10 is syntenic to human chromosome 6q. A large number of human non-familial malignant melanomas display rearrangements in this same region of human chromosome 6 [66,67]. We identified the deleted 70kb host region to be part of intron 3 of the gene encoding the metabotropic glutamate receptor 1 protein, mGlu_1_. In order to definitively demonstrate that *GRM1* has a direct etiological role in melanoma development in our model system, we generated a new transgenic line using wild-type mouse *Grm1* cDNA under a melanocyte-specific promoter, dopachrome tautomerase (Dct). Indeed, pigmented tumors developed in the founder and subsequent progeny of this new transgenic line (line E) [16,19]. These results unequivocally demonstrated that the introduction of *Grm1* cDNA alone to melanocytes was sufficient to induce melanoma development *in vivo* with 100% penetrance. More recently, Ohtani and co-workers have validated our findings. They demonstrated that following the activation of a conditional *GRM1* transgene, their transgenic mice develop pigmented lesions on the ears and tail with 100% frequency at 52 weeks [68]. 

Identification of aberrant mGlu_1_ expression in melanocytes being the causative agent in melanoma development in TG-3/E transgenic lines directed us to extend our studies to human melanoma. Initially, we demonstrated mGlu_1_ expression in seven out of 19 human melanoma biopsies and 12 out of 18 melanoma cell lines [19]. To date we have tested more than 120 human melanoma biopsies and 25 human melanoma cell lines, and found about 60% of these samples express mGlu_1_ at levels of both RNA and protein. In addition, a report by Nishigori and colleagues showed mGlu_1_ expression in 80% (49/61) of melanoma tissue samples consisting of superficial spreading, nodular, lentigo maligna, acral lentiginous and metastatic melanomas [69]. mGlu_1_ expression was also detected in 33% (6/18) of common, blue and Spitz nevi. mGlu_1_expression was also observed in 75% (6/8) of human melanoma cell lines and 50% (2/4) of nevus derived cell lines while none of the normal melanocytes assayed were positive [69]. These additional data strongly suggest that a better understanding of the regulation of mGlu_1_ expression in melanocytes at the molecular level will likely identify new target(s) and contribute to the design of more effective treatments for this disease.

**Figure 1:**
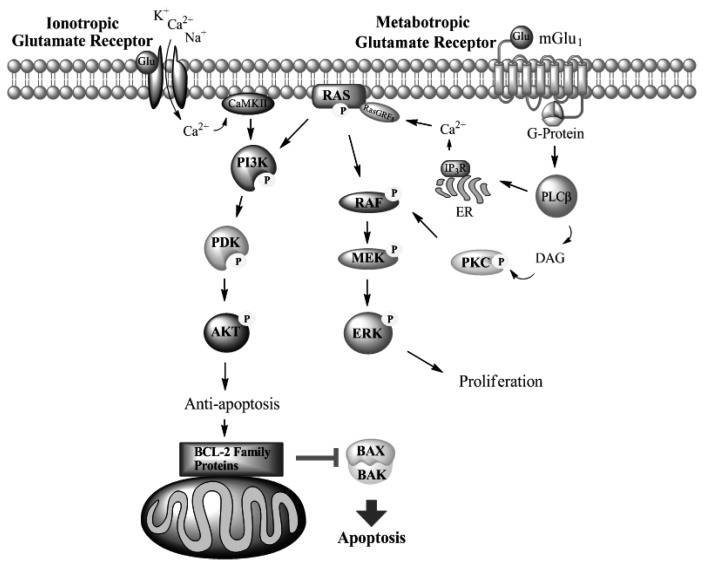
Depicts our current hypothesis on how mGlu_1_ and its downstream effectors likely promote melanoma growth.

To investigate the mechanism by which the expression of mGlu_1_ in melanocytes results in melanoma formation, we analyzed several human melanoma cell lines to assess whether the ectopically expressed receptor was functional in human melanocytes [38]. C8161 and WM239A are two human melanoma cells that express mGlu_1_. The functionality of mGlu_1_ in these cells was shown by the accumulation of the second messenger, inositol-1,4,5-triphosphate (IP_3_) when the receptor is stimulated by the mGlu_1_-agonist, Quisqualate. The specificity of Quisqualate-induced build-up of IP_3_ was demonstrated by the absence of IP_3_ accumulation when the cells were pre-treated with mGlu_1_-antagonists, LY367385 or BAY36-7620 [38]. Earlier, using several mouse tumor cell lines derived from TG3 tumors, we showed that the MAPK pathway, a key signaling pathway in human melanoma, was activated by Quisqualate as indicated by enhanced ERK phosphorylation while pre-treatment of these cells with the antagonist LY367385 abolished Quisqualate-induced ERK activation [70]. Similar results were detected in mGlu_1_-expressing human melanoma cells [38]. These results demonstrated that the receptor, mGlu_1_ is functional in mGlu_1_-expressing human melanoma cells and that activation activates signaling components that promote cell proliferation as depicted in Figure 1 below.

Previous reports by others [71,72] indicate that glutamate receptor antagonists inhibit cell proliferation. We investigated whether the competitive mGlu_1_ antagonist LY367385 or the non-competitive mGlu_1_ antagonist Bay 36-7620 could inhibit the proliferation of mGlu_1_ positive C8161 human melanoma cells. Both antagonists suppressed the growth of the mGlu_1_ positive melanoma cells more than the control human embryonic kidney (HEK) and primary human embryonic melanocytes (HEM) cells which do not express the receptor [38]. The non-competitive antagonist Bay 36-7620 was however more effective than the competitive antagonist LY367385 [38]. These observations prompted us to perform follow up experiments to identify other compounds that could suppress the growth of melanoma reliant on glutamate and mGlu_1_ for growth. 

In an earlier study with a transgenic line ectopically expressing hepatocyte-growth factor/scatter factor, a correlation between oncogenesis and high autocrine activity of hepatocyte-growth factor/scatter factor (the ligand) and its receptor (receptor tyrosine kinase, Met) was described [73]. Based on these observations, we were interested to know if aberrant expression of mGlu_1_ in melanocytes may promote autocrine activity by increasing the availability of the ligand, glutamate. Levels of extracellular glutamate were evaluated in several human melanoma cell lines and they all exhibited excess extracellular glutamate in comparison to the control human embryonic kidney cells, HEK [38]. In light of this observation, it is not surprising that the competitive mGlu_1_ antagonist, LY367385 was less effective when used to suppress mGlu_1_ function. LY367385 competes with the ligand, glutamate, for the same receptor binding site in mGlu_1_ expressing cells. Thus, the enhanced levels of extracellular glutamate in human melanoma cells render the competitive antagonist less effective [38]. We also used genetic means to silence the endogenous *GRM1* gene. This was done by introducing exogenous dominant negative *GRM1* constructs in human melanoma cell lines that resulted in a reduction in melanoma cell growth [38]. Taken together, these results demonstrated that ablation of mGlu_1_ receptor function by pharmacological or genetic means yielded a decrease in tumor cell growth.

Glutamate is the most abundant excitatory neurotransmitter in CNS. Regulation to maintain appropriate levels of glutamate release is required for normal neuronal function such as learning, memory or plasticity of the brain. Irregular glutamate release has been implicated in various neuro-pathological conditions from stroke to chronic neuro-degenerative disorders such as Alzheimer’s disease, Huntington’s disease, and amyotrophic lateral sclerosis (ALS). Riluzole is the only Food and Drug Administration (FDA) approved drug for the treatment of ALS, where it has been shown to reduce the progression of the disease [74,75,76]. The mode of action of Riluzole is largely unknown, but has been attributed to the inhibition of glutamate release. The ability of Riluzole to block the release of the ligand for mGlu_1_, glutamate, mimics its function as a putative antagonist of glutamate receptors thus interfering with intracellular events that follow stimulation of the receptor. Based on these properties, we predicted that it would be effective in reducing mGlu_1_-positive human melanoma cell growth. We demonstrated suppression of melanoma cell proliferation in the presence of Riluzole in cultured cells *in vitro* and validated the results with xenografts *in vivo* [38]. 

The translation of our preclinical laboratory findings into the clinic began with a Phase 0 trial of Riluzole in patients with resectable stage III and IV mGlu_1 _positive melanomas**. **Phase 0 trials have been recommended by the FDA as proof of mechanism studies for signal modulating agents. Riluzole is an FDA approved drug for the treatment of ALS [74]. The maximum-tolerated daily dose in humans is 200 mg/day [74,77,78,79], and therefore a dose-determination phase I trial was not necessary. Pretreatment biopsies were obtained from patients enrolled in the trial, received 200 mg/day of oral Riluzole for two weeks and then underwent resection of their residual tumors. Patient dosing for two weeks was chosen because this represents approximately 7 half-lives of the drug after it reaches steady state [74,77,78,79]. We also obtained pre-and post-treatment PET (positron emission tomography) scans to evaluate the overall metabolic activity of the tumors and how this activity changes with inhibition of mGlu_1_ mediated signaling. 12 patients were enrolled in this Phase 0 trial with 11 successfully completing the protocol with little toxicity noted. One patient was removed for grade III toxicity (dizziness) that resolved upon stopping the drug. Even though clinical responses with just two weeks of Riluzole administration were unexpected, three patients had obvious shrinkage of their tumors with. Four patients had significant decreases in Standardized Uptake Value (SUV) intensity on the post-treatment PET scans as compared to the pre-treatment scans. In three cases, some of the multiple nodal and cutaneous metastases completely resolved and in all four cases, the post treatment PET scans showed tumors with significant decreases in SUV intensity. Four of the patients had stable disease and the remaining patients had progressive disease at the end of the trial. Possible modulation of the glutamatergic pathway by Riluzole treatment was assessed by examining levels of phosphorylated ERK and Akt in pre- and post-treatment samples. Dramatic reduction in the phosphorylation of ERK was detected in four patients who had shown clinical responses while a decline in levels of phosphorylated Akt was observed in three patients that exhibited clinical responses to Riluzole [80]. With the unexpected success of the Phase 0 trial, we have proceeded to a therapeutic Phase II randomized trial involving more patients with stage III melanoma. This trial will determine the response rate, durability of response, and long-term toxicity of oral Riluzole in patients with advanced melanoma and also examine potential biological correlates of response to Riluzole therapy. 

Despite the unexpected remarkable therapeutic outcome in the Phase 0 trial with Riluzole, we realize that cancer patients have a relatively heterogeneous genetic profile reflecting the general population; therefore it is likely that single agent Riluzole will not prove effective in some patients as we proceed through clinical trials. We have begun to explore combinatorial therapies that include Riluzole as one of the components with known inhibitors with suppressive activities against constitutively activated signaling pathways in melanomas. We choose to start with the RAF inhibitor Sorafenib because of its effects on RAF signaling and it’s well known toxicity profile *in vivo* [81]. We treated two mGlu_1_-expressing human melanoma cell lines with Riluzole in combination with Sorafenib in cell proliferation/viability *in vitro* MTT (methylthiazolyldiphenyl-tetrazolium bromide) assays and *in vivo* xenograft assays. We demonstrated that combination of Riluzole and Sorafenib led to a considerable decrease in the number of viable cells in human melanoma cell lines *in vitro* and a reduction in the tumor volume in xenografts *in vivo* than that achieved with either single agent alone [82]. These results propose that the combination of Riluzole and Sorafenib would be a reasonable combinatorial therapy for the treatment of patients with advanced melanoma and will be investigated in an upcoming clinical trial. 

## Glutamate Receptors in Other Cancers

Oncogenic potential of glutamate receptors has been recognized with the detection of various subtypes of glutamate receptors in a variety of cancer cell lines and tumor samples including neuroblastomas, gliomas, medullablastomas, melanoma and osteosarcomas [19,71,83,84,85,86]. Neuronal tumors show a great predisposition to the expression of various metabotropic and ionotropic glutamate receptors [84,85,87]. In gliomas, expression of ionotropic and metabotropic glutamate receptors has been described. Involvement of one of these receptor in gliomas was demonstrated by the use of short hairpin RNAs (shRNAs) to silence the expression of the AMPA type GluR1 in glioma cells, which resulted in inhibition of cell growth [71,84,88]. Studies have also identified the expression of mGlu_4_ in colorectal cancer [89,90]. mGlu_5_ in oral squamous cell carcinoma [91], the NMDA receptor type 2B in gastric cancer [92] and the *N*-methyl-D-aspartate receptor (NMDAr) in prostate cancer [93]. There is evidence for clinical significance of the expression of these glutamate receptors; for example the expression of mGlu_5_ in oral squamous cell carcinoma [91] has been shown to correlate with increased survival of the patients while an inverse correlation has been noted between the expression of mGlu_4_ in medullablastoma and increasing tumor severity and recurrence after therapy [87]. Thus the clinical significance of the expression of each glutamate receptor subtype identified in these cancers needs to be established to better guide potential therapy.

## Conclusions

Our understanding of the divergent roles of the neurotransmitter glutamate, and its receptors has increased exponentially in the last decade. The unexpected but significant role of these receptors in the development of various neoplasms has generated new ideas for the identification of novel drug targets and congruent therapies. In metastatic melanoma, the need for new drugs is driven by the rising number of diagnosed cases and morbidity coupled with chemoresistance and a lag in drug development. Dacarbazine, a drug approved almost 30 years ago, is considered the reference single agent for the management of advanced melanoma, with objective responses in approximately 13–20% of patients, most of them being partial and a few complete responses [94,95]. Interferon (IFN)-α has remained the corner stone of adjuvant therapies for patients with stage II and stage III disease with high doses shown to prolong disease-free and overall survival [96,97]. Targeting know melanoma causative genes and mutations might yield greater success in the development of clinically viable therapies. The identification of a novel oncogene, *GRM1*, which plays a causative role in over 60% of melanoma cases, is especially significant in melanoma drug development. Also, the understanding that glutamate the ligand for mGlu_1_ contributes to aberrant melanocyte growth in cells harboring the receptor through an autocrine loop has led to the utility of a glutamate release inhibitor, Riluzole as a potential anti-melanoma drug. The success of our current clinical trials with single agent Riluzole and the combination of Riluzole with Sorafenib would be beneficial to patients with mGlu_1_ positive melanomas regardless of common mutations like BRAF V600E. Given the critical role of glutamate receptors in cancer development, more resources are needed to advance rational designs of glutamate receptor antagonists for therapies. 
